# Multivariate-Time-Series-Driven Real-time Anomaly Detection Based on Bayesian Network

**DOI:** 10.3390/s18103367

**Published:** 2018-10-09

**Authors:** Nan Ding, Huanbo Gao, Hongyu Bu, Haoxuan Ma, Huaiwei Si

**Affiliations:** School of Computer Science and Technology, Dalian University of Technology, Dalian 116024, China; huanboliuyue@mail.dlut.edu.cn (H.G.); bhy650@mail.dlut.edu.cn (H.B.); haoxuan19950916@mail.dlut.edu.cn (H.M.); sihuaiwei@mail.dlut.edu.cn (H.S.)

**Keywords:** multivariate-sensing time-series, anomaly detection, hierarchical temporal memory, bayesian network

## Abstract

Anomaly detection is an important research direction, which takes the real-time information system from different sensors and conditional information sources into consideration. Based on this, we can detect possible anomalies expected of the devices and components. One of the challenges is anomaly detection in multivariate-sensing time-series in this paper. Based on this situation, we propose RADM, a real-time anomaly detection algorithm based on Hierarchical Temporal Memory (HTM) and Bayesian Network (BN). First of all, we use HTM model to evaluate the real-time anomalies of each univariate-sensing time-series. Secondly, a model of anomalous state detection in multivariate-sensing time-series based on Naive Bayesian is designed to analyze the validity of the above time-series. Lastly, considering the real-time monitoring cases of the system states of terminal nodes in Cloud Platform, the effectiveness of the methodology is demonstrated using a simulated example. Extensive simulation results show that using RADM in multivariate-sensing time-series is able to detect more abnormal, and thus can remarkably improve the performance of real-time anomaly detection.

## 1. Introduction

**Figure 1:**
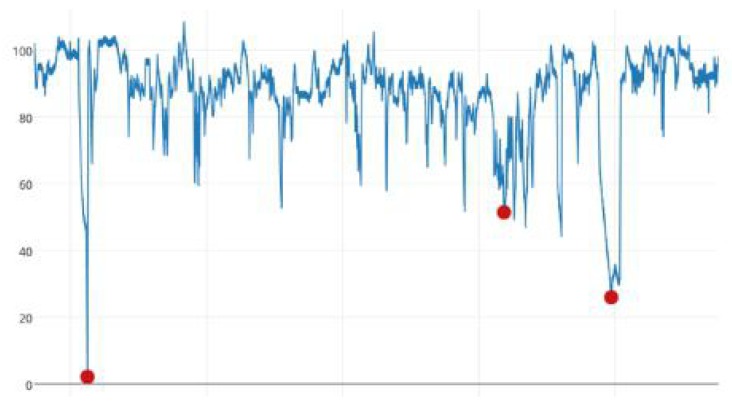
Spatial anomalies and temporal anomalies.

The system condition monitoring associated with virus invasion, failed sensors, and improperly implemented controls plagues many automated information system, such as wireless sensor networking, vehicular networking, and industrial system. The real-time anomaly detection for system condition monitoring has significant and practical applications, which uses the information coming in real-time from different sensors and other condition information sources and tries to detect possible anomalies in the normal condition and behaviour expected of its devices or components. Anomalies can be spatial, known as spatial anomalies, which means the values are outside the typical range like the 1st and 3rd anomalies in Figure 1, which is time series data collected from temperature sensors in a factory (The anomaly in Figure 1 is marked in red). Also anomalies can be temporal, known as temporal anomalies.The values are not out of typical range but the sequence generated from which is anomalous, like the second anomaly in Figure 1 [1].

At present, there is extensive work on anomaly detection techniques looking for individual objects that are different from normal objects. Some of them mainly focus on the anomaly detection in univariate time series (UTS). Holt-Winters is one of the anomaly detection methods which can detect spatial anomalies and it is widely implemented for commercial applications [2]. The Skyline project provides an open-source implementation of a number of statistical techniques for anomaly detection in streaming data [3]. Autoregressive Integrated Moving Average Model(ARIMA) is a general technique for modeling temporal data with seasonality which can perform temporal anomaly detection in complex scenarios [4]. Bayesian change point methods are a nature method which segments time series and can be used for online anomaly detection [5,6]. Twitter released its own open-source anomaly detection algorithms for time series data, it is capable of detecting spatial and temporal anomalies, and has gotten a relatively high score in the Numenta Anomaly Benchmark (NAB) scoring mechanism [7,8]. In addition, there are a number of model-based methods applied to specific fields, examples include detection for cloud data center temperatures [9], ATM fraud detection [10], anomaly detection in aircraft engine measurements [11], and some excellent work based on an accurate forecasting solutions with application to the water sector [12,13] so on.

However, in a complex system, compared to the anomaly detection in UTS, it brings richer system information using multivariate time series (MTS), which are captured from the sensors and condition information sources. For the methods of anomaly detection in MTS, there are two main categories, one is to use the method of detecting anomaly after dimension reduction. Existing dimensionality reduction methods, such as PCA (Principal component analysis) dimensionality reduction method used for MTS and the linear dimensionality reduction method used for MTS based on common principle component analysis, are processing the principle time series according to the anomaly detection method for UTS [14]. Another method is to take the sliding window as a tool for dividing MTS, and then detect the subsequences. For example, one method is to calculate each covariance matrix of subsequence based on Riemannian manifolds and make the covariance matrix as the descriptor, Riemannian distance as the similarity measure to calculate the distance between the covariance matrix. And it can show the existence of abnormal intuitively through the distribution of the covariance matrix and its visualization [15,16]. These methods could meet the requirements for anomaly detection in MTS, but due to the lack of consideration of the inherent relevance in MTS, actual effect and accuracy are required to be improved. In order to improve the efficiency of the algorithm, this paper introduces the concept of health factor α which means whether the system is running well enough or not. Introducing health factor can greatly reduce the cost of RADM in a healthy system.

In this paper, we propose a real-time anomaly detection algorithm in MTS based on Hierarchical Temporal Memory (HTM) and BN (Bayesian Network). The remainder of the paper is structured as follows. Section 2 provides a brief introduction to the performance problems and scenario of anomaly detection in MTS, while Section 3 is dedicated to presenting the methodology of HTM. The proposed RADM is discussed in Section 4. Then, in Section 5, to demonstrate the principles and efficacy, results are presented comparing RADM with HTM. Finally, in Section 6, concluding remarks and possible extensions of the work are discussed.

The contributions of this paper can be summarized as follows:

1. This paper proposes an anomaly detection framework based on multivariate-sensing time-series data to achieve real-time anomaly detection, and improve the performance of anomaly detection.
2. This paper introduces the concept of health factor α to describe the health of the system, and further more, greatly improve the detection efficiency of health systems.
3. RADM combines HTM with naive Bayesian network to detect anomalies in multivariate-sensing time-series, and get better result compared with the algorithm just work in univariate-sensing time-series.

## 2. Performance Problems and Scenario

**Figure 2:**
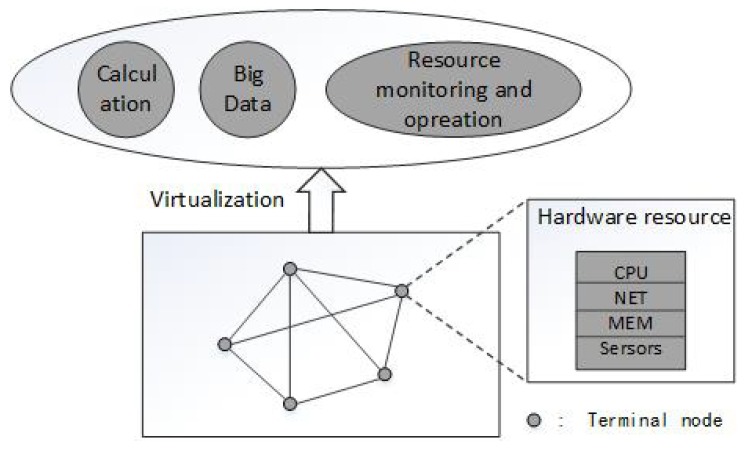
The structure of Cloud Platform.

It has been proved that cloud computing could help by offering on-demand and scalable storage, as well as processing services that can scale to the requirements of IoT and automated industry system. In these applications, Cloud Platform virtualizes physical devices (hosts, switches, storage and sensors, etc.) based on virtualization, and by virtualizing the infrastructure engine integrates the virtual computing, storages and networks resources into a centralized resource pool. And this resource pool is delivered to the upper application system in an automated and self-service way [17,18]. The structure of Cloud Platform is shown in Figure 2.

At present, related works utilize system parameters to characterize the real-time state of the system, and implement the real-time detection for its state. In order to perform the anomaly detection in MTS, based on the real-time monitoring cases of the system states of terminal nodes in Cloud Platform, we virtualize the node system state into CPU, NET and MEM parameters, as shown in Figure 2, and use them as anomaly detection sequences to characterize the system state. We describe the system state as follow:

(1) S(t)=(X(t),Y(t),Z(t))

where, X(t) represents the time series data of CPU, Y(t) is the time series data of NET, and Z(t) means the time series data of MEM. Since NET accounts for a large proportion in the reasons of system anomalies, we set NET as the principle time series, and it is utilized in anomaly detection in UTS which is used as a comparison in the experiment.

## 3. UTS and HTM

### 3.1. HTM Cortical Learning Algorithm

HTM is a machine learning method designed to capture the structure and operating characteristics of the new cerebral cortex. HTM is essentially a memory-based system, and the HTM network relies on a large number of pattern sequences stored in it, and it is trained by a large number of time series data. The HTM network consists of hierarchical regions, and each of the regions represents a level in the hierarchy. Hierarchy information will continue to converge with the rise of the level, and also diverge with the decline of the level. The spatial pooler and the temporal pooler are used in HTM to learn and predict from the input data. The HTM region contains a columnar region consisting of cells, and through the spatial pooler each cell produces a sparse distributed representation, which is used to record the active state of cells. Then the temporal pooler can discover and learn pattern from the list of active columnar regions calculated from the spatial pooler, and serialize it for prediction [19].

### 3.2. Anomaly Detection in UTS Based on HTM

HTM formalizes the process as follows. It lets the vector $x_{t}$ represent the state of a real-time system at time *t*. The HTM model receives a continuous stream of inputs:

(2) $$\cdots ,x_{t-2},x_{t-1},x_{t},x_{t+1},x_{t+2},\cdots$$

**Figure 3:**
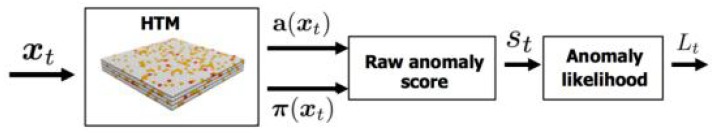
The primary functional steps in HTM algorithm.

In practical applications, the statistics of the system can change dynamically, real-time learning and training are needed to perform the detection of new anomalies. HTM is a learning algorithm which appears to match the above constraints and has been shown to work well for prediction tasks [20], however, it does not directly output the anomaly score. In order to perform anomaly detection, two different internal representations are used in the HTM. As shown in Figure 3  [1]. Given an input $x_{t}$, the vector a ($x_{t}$) is a sparse binary code representing the sparse distributed representation of the current input. Also an internal state vector π ($x_{t}$) is used to represents a prediction for a ($x_{t+1}$), that is, a prediction of the next input $x_{t+1}$. The prediction vector incorporates inferred information about current sequences, which is dependent on the current detected sequence and the current inferred position of the input in the sequence, and this means different inputs will lead to different predictions.

However, a ($x_{t}$) and π ($x_{t}$) do not directly represent anomalies. In order to create a robust anomaly detection system, HTM introduces two additional steps. Firstly a raw anomaly score is computed from the two sparse vectors. Then HTM computes an anomaly likelihood value which is thresholded to determine whether the system is anomalous.

#### 3.2.1. Computing the Raw Anomaly Score

In order to measure the deviation between the predicted input and the actual input of the model, a raw anomaly score is computed from the intersection between the predicted and actual sparse vectors. At time t the raw anomaly score $s_{t}$ is given as:

(3) $$s_{t}=1-\frac{\pi \left(x_{t-1}\right)\cdot a\left(x_{t}\right)}{\left|a(x_{t})\right|}$$

The raw anomaly score will be 0 if the current input is perfectly predicted, 1 if it is completely unpredicted, or somewhere between 0 and 1 according to the similarity between the input and the prediction.

Because of the continuous learning nature of HTM, the changes of the underlying system can be handled gracefully. If there is a shift in the behavior of the system, the anomaly score will be high at the point of this shift, however, as the HTM model adapts to the “ new normal ”, the anomaly score will degrade to zero automatically [1].

#### 3.2.2. Computing the Anomaly Likelihood

HTM uses the distribution of the anomaly scores to calculate the anomaly likelihood which defines how anomalous the current state is based on the prediction history of the HTM model. HTM maintains a window of the last *W* raw anomaly scores and models the distribution of the anomaly scores as a rolling normal distribution, where the sample mean $\mu_{t}$ and variance $\sigma_{t}$ are continuously updated from the previous anomaly scores, Using k as the number of windows, as follows:

(4) $$\mu_{t}=\frac{\sum_{i=0}^{i=W-1}s_{t-i}}{k}$$

(5) $$\sigma_{t}^{2}=\frac{\sum_{i=0}^{i=W-1}{\left(s_{t-i}-\mu_{t}\right)}^{2}}{k-1}$$

Then HTM evaluates a recent short-term average of the anomaly scores, and thresholds the Gaussian tail probability (Q-function [21]) to decide whether or not to declare an anomaly. The anomaly likelihood is defined as the complement of the tail probability:

(6) $$L_{t}=1-Q\left(\frac{\tilde{\mu_{t}}-\mu_{t}}{\sigma_{t}}\right)$$

where:

(7) $$\tilde{\mu_{t}}=\frac{\sum_{i=0}^{i=W^{\prime }-1}s_{t-i}}{k}$$

$W^{\prime }$ is a window for a short-term moving average, where $W^{\prime }$ << *W*. HTM sets the threshold Lt, if it is very close to 1, an anomaly will be reported:

(8) $$anomaly\equiv L_{t}\geq 1-\epsilon$$

HTM sets *Q* as an array of the tail probability for the standard normal distribution, which preserves the probability corresponding to the mean. In order to facilitate the calculation, HTM divides the range of values of μ in the normal distribution[σ, 3.5σ] into 70 intervals equidistantly, so that it sets the tail probability to 71 values and puts them into an array *Q*, as shown in Table 1.

In the process of thresholding $L_{t}$, thresholding the tail probability makes the number of alerts have an inherent upper limit. In addition, since ϵ is very close to 0, it would be unlikely to get alerts with probability much higher than ϵ, which also imposes an upper limit on the number of false positives.

## 4. MTS and RADM

In order to meet the requirements for anomaly detection in MTS and consider the inherent relevance between MTS, we utilize a BN model to analyze the validity of MTS, and propose a corresponding anomaly detection algorithm in MTS, RADM.

### 4.1. Bayesian Network Analysis

#### 4.1.1. Bayesian Network

BN is a combination of Graph Theory and Probability Theory, and consists of a directed acyclic graph and the probability of each node. This directed acyclic graph is called the BN structure, the probability of each node is called the BN parameter, BN gets the BN parameters through structural learning or parameter learning. The basic formula of the graphical network is the Bayesian formula [22]. According to the Bayesian formula, a sample *x* is known, and the probability that this sample belongs to the category *y* is:

(9) $$P\left(y|x\right)=\frac{P(y)P(x|y)}{P(x)}$$

where *x* is the sample characteristic variable, that is, the known quantity. According to the Total Probability Theorem, expansion is shown as follows:

(10) $$P\left(y=c_{k}|x\right)=\frac{P\left(x_{1},x_{2},\cdots ,x_{i}|y=c_{k}\right)P\left(y=c_{k}\right)}{\sum_{k}P\left(y=c_{k}\right)P\left(x_{1},x_{2},\cdots ,x_{i}|y=c_{k}\right)}$$

where $c_{k}$ represents the *k*-th class of *y* and *i* is the number of characteristic variables of *x*, $x_{i}$ is the value corresponding to the *i*-th characteristic variable. Let *M* be the characteristic variable dimension of *x* (i.e., the total number of characteristic variables). If characteristic variables in the sample *x* are independent of each other, the above formula can be simplified as:

(11) $$P\left(y=c_{k}|x\right)=\frac{\prod_{i=1}^{M}P\left(x_{i}|y=c_{k}\right)‗P\left(y=c_{k}\right)}{\sum_{k}P\left(y=c_{k}\right)\prod_{i=1}^{M}P\left(x_{i}|y=c_{k}\right)}$$

#### 4.1.2. Naive Bayesian Network in MTS

We choose to build a Naive Bayesian classifier with multivariate parameters. Naive Bayesian is insensitive to missing data, and it has minimal error rate compared to other classification methods [23].

**Figure 4:**
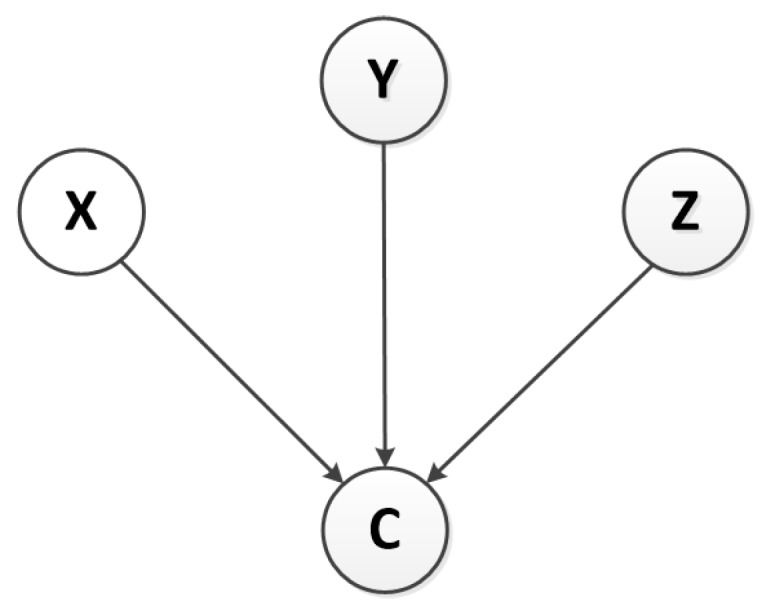
The Topology of Bayesian Network.

The topological structure of the Naive Bayesian model is shown in Figure 4, *C* represents the classification mark, that is, the output sequence, *C* = {normal, anomaly}. We mark the state variables of X(t), Y(t), Z(t) as *X*,*Y*, and *Z* respectively, that is, the discrete values corresponding to the anomaly likelihood of the ternary time series. Because the nodes of the Naive BN are independent of each other, its classifier satisfies the Equation (11).

For the same group of state variables [*X*,*Y*,*Z*], the denominator of the formula is the same, so only the numerator is needed to be compared and we get:

(12) c(x)=argmaxP(X,Y,Z|c)P(c)

When using a set of [*X*, *Y*, *Z*] data as a training sample (training set), the following Bayesian formula is used to calculate the posterior distribution of the target variable:

(13) $$c\left(x\right)=argmax\prod_{i=1}^{3}P\left(a_{k}|c\right)P\left(c\right)$$

where $a_{k}$ represents three attribute variables[*X*, *Y*, *Z*], so *k* can be the value of 1,2,3.

The following formula is used to calculate P(c):

(14) $$P\left(c\right)=\frac{\sum_{i=1}^{n}I\left(c=c_{j}\right)}{n}$$

where, *n* is the number of training samples, $c_{j}$ marks the *j*-th category of *c*, *I* (*c* = $c_{j}$) is the instruction function, when the equation in brackets is true, its value is 1, otherwise 0. Then it is assumed that the *k*-th characteristic in the three characteristics has *l* values, and one of the values is assumed to be $a_{jl}$, the following formula is used to calculate *P* ($a_{k}$|*c*):

(15) $$P\left(a_{k}|c\right)=\frac{\sum_{i=1}^{n}I\left(x_{i}^{j}=a_{jl},c=c_{j}\right)}{\sum_{i=1}^{n}I\left(c=c_{j}\right)}$$

After the above calculation, we can get the probability that a set of eigenvalues belongs to a class and then perform the classification.

### 4.2. RADM

**Figure 5:**
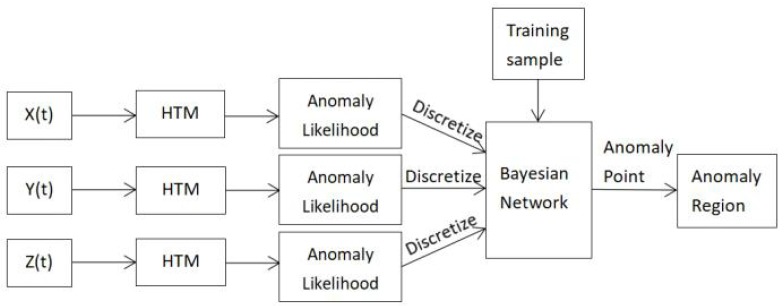
RADM flowchart.

Based on the above HTM algorithm and Naive Bayesian model, we design a real-time anomaly detection algorithm in MTS, RADM. The anomaly detection flowchart of RADM is described in Figure 5.

(1) HTM Algorithm

The ternary sequences X(t), Y(t) and Z(t) need to be detected separately, three HTM networks are used to study and predict three sequences, we get the anomaly likelihood through HTM algorithm, and a list of anomaly likelihood is composed of the anomaly likelihood in three sequences. The specific process can be found in Section 3.

(2) Discretization

Because in the calculation of anomaly likelihood the values of the anomaly likelihood are also divided into 70 intervals, in order to facilitate the study and reduce the amount of data and calculation of training sample in parameter learning, we need to discretize the anomaly likelihood. By analyzing the likelihood data we know that the anomaly likelihood of each variable is related to the standard normal distribution and the range of the low anomaly likelihood is large but the effect to the anomaly detection is negligible. After the experimental tests, combined with the short-term sliding average we use the equivalent interval method to get the threshold intervals and the discrete values, as shown in Table 2.

(3) Bayesian Network

We use the method of parameter learning to build Naive BN. After we know the Naive Bayesian structure, the corresponding weights are assigned to each time series according to the prior knowledge. Since NET is the principle time series in this experiment, we need to increase the weight of NET when training BN, and then builds the training sample based on these weights to train BN. We use Matlab to implement BN and after the discretization the junction tree method is used to infer the anomaly classification, so that we get the list of anomaly regions.

The relevant pseudo-code of RADM is shown as follow Algorithm 1: 

**Algorithm 1:** RADM.1: Input: S(t); 2: Output: ARmuti(t);// Anomaly regions in MTS 3: **while** 1 **do** 4: ALx(t) = HTM(S(t).X(t)); // The list of anomaly likelihood in X (t) through HTM 5: ALy(t) = HTM(S(t).Y(t)); 6: ALz(t) = HTM(S(t).Z(t)); 7: D(X,Y,Z) = Discrete(ALx(t),ALy(t),ALz(t)); // Discretization 8: ARmuti(t) = Bayesian(D);// BN inference 9: **end while**

Since each exception likelihood obtained by HTM corresponds to a point in a time series, in the actual test, there will be continuous anomaly points. It is obviously unreasonable to judge the number of anomalies based on anomaly points. Therefore we use an anomaly region to represent an anomaly. The anomaly region is divided according to the continuous situation between the anomaly points, the formal definition is as follows:

If the distance between an anomaly point A1 and another anomaly point A2 is less than the division distance S, then we put A1 and A2 into the same anomaly region, on the contrary, they belong to different anomaly regions. The division distance S is also called the division window.

(4) Health Factor

So as to improve the efficiency of the algorithm, this paper introduces the concept of health factor α, which is defined as Formulas (16) and (17). S${}_{n}$ is the anomaly score calculated by HTM. Health factor means whether the system is running well enough or not. The reason for introducing health factor is that the ratio of anomalies in different systems is different. For a system with extremely low probability of anomalies in daily operation, if we import all data into the Naive Bayesian model for joint detection, it will waste a lot of system resources and also affect the temporal performance of the algorithm. In this paper, only when α is higher than the threshold θ, the data is imported into the Naive Bayesian model for joint detection, which can effectively improve the temporal performance of the algorithm. The threshold θ can be dynamically selected between 0 and 1 depending on the system state. When θ is equal to 0 (θ = 0), it means that all the data is imported into the Naive Bayesian model for joint determination, which corresponds to the condition where the system state is extremely unhealthy. When θ is equal to 1 (θ = 1), it represents the system is completely normal, and no matter what value of α is, it is not necessary to enter the Naive Bayesian model for joint determination.

(16) $$H=(S_{1},S_{2},S_{3},\cdots ,S_{n})$$

(17) $$\alpha =\left||H|\right|=\sqrt{{\left(S_{1}-\bar{S_{1}}\right)}^{2}+{\left(S_{2}-\bar{S_{2}}\right)}^{2}+\cdots +{\left(S_{n}-\bar{S_{n}}\right)}^{2}}$$

## 5. Experimental Simulation and Performance Analysis

Through experiments, we verify the effect of anomaly detection in MTS based on RADM, simultaneously, adding the experiment of anomaly detection in UTS based on HTM algorithm as a comparison. By comparing the results of these two experiments, we analyze the advantages of RADM for anomaly detection, and how the anomaly detection changes from UTS to MTS.

### 5.1. Simulation Environment and Parameter Settings

As for the implementation of HTM algorithm, we select the htm.java code library published by Numenta on Github in 2017 [24], runtime environment is Java (TM) SE Runtime Environment 1.8.0. We use FullBNT-1.0.4 as Bayesian tool, compiler environment is Matlab 2016b.

In the anomaly detection in UTS, window $W^{\prime }$ for sliding short-term average should be much smaller than the sliding time window *W*, in our experiment, *W* is set to 100 and $W^{\prime }$ is set to 5, anomaly threshold is set to 0.99998220767. In addition, we use an anomaly region to represent an anomaly, the size of the division window of the anomaly region is set to 10. In order to avoid the influence of different health factor α on the performance of the algorithm, experiment makes α = 0, that is, the overall data in the dataset needs to enter the Naive Bayesian model for analysis.

### 5.2. Data Sample Description

Numenta sets the Numenta Anomaly Benchmark (NAB) which is an open-source framework, to compare and evaluate algorithms used to detect anomalies in real-time streaming data. Its NAB dataset contains real world tag data from multiple domains, which includes the normal time-series data and the labeled anomalous time-series data, and it has been proved to be effective in the assessment of the anomaly detection in UTS [7].

In order to perform the real-time anomaly detection in MTS, we refer to the NAB dataset and its requirements, by means of stress-testing approaches we let X(t), Y(t) and Z(t) contain anomalous subsequences, and with the help of the open-source tool sigar1.6.4 java version we program and collect X(t), Y(t) and Z(t) in the computer. We use them as data samples input into RADM algorithm.

### 5.3. Performance Analysis and Comparison

#### 5.3.1. Relevance Analysis

RADM can detect the anomalies in the time series effectively through the multi-parameter auxiliary judgment. For a complex system, the variables are often interrelated, this relationship can be reflected in the MTS. And BN has the advantage of dealing with uncertainties, it can show this relationship intuitively and simply in the form of probability.

**Figure 6:**
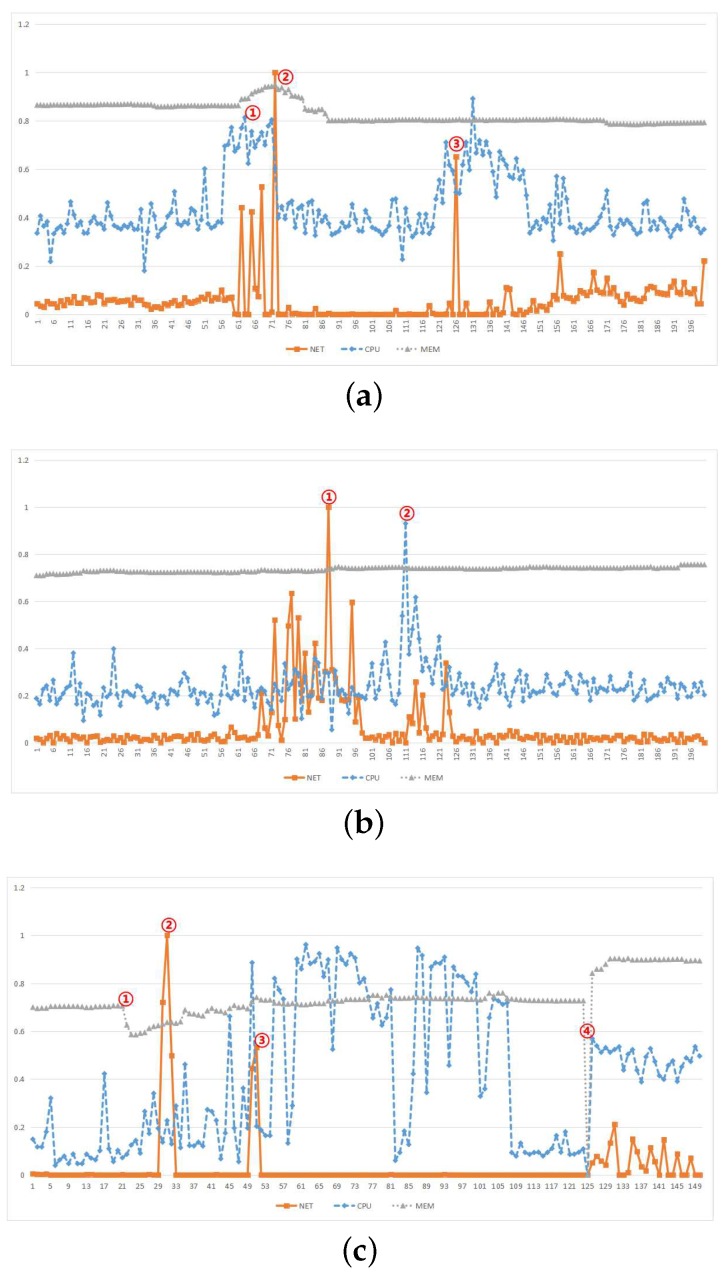
Analysis of the relevance between different dimensions: (**a**) Establish relevance based on MEM, (**b**) Independent CPU and NET, (**c**) Mixed situation.

As shown in Figure 6, from the three points in Figure 6a the relevance of the data is reflected, we can see that in the vicinity of these points, the NET changes will cause changes of CPU and MEM, which means when an anomaly occurs in the associated data, other time series also have a relatively large probability of anomaly. The two points in Figure 6b reflects the necessity of anomaly detection in MTS. Shortly after the peak of NET occurs, the CPU reaches peak, making the range of anomaly regions increase, which is not possible for anomaly detection in UTS. From the Figure 6c we can see when only the NET time series is detected, the range of the anomaly region is between the 2nd and 3rd points, but because of the detection of other anomaly time series, the range of anomaly region is extended to the scope of the 1st and 4th points, which also enables that the anomaly can be detected in advance. The expansion of the anomaly region helps to understand the anomaly information and make more comprehensive decisions.

Definitely, the anomaly detection in UTS is the basis of RADM. However, as the dimension increases, the MTS can play a greater role in anomaly detection in a complex system and reflect the relevance between data, and this relevance is the embodiment of data validity.

#### 5.3.2. Accuracy Analysis

We perform anomaly detection in UTS based on HTM algorithm and anomaly detection in MTS based on RADM separately. The two experiments use the same data, which is, when performing anomaly detection in UTS, only detecting Y(t) in the MTS. And ternary time series need to be detected in the anomaly detection of RADM.

The experimental results are shown in Table 3. It can be seen from the 1st, 4th group data that RADM can detect more anomaly regions than the anomaly detection in UTS. However, the 5th, 6th group data show that the anomaly regions detected by RADM is less than the anomaly detection in UTS, which does not mean the capability of RADM is low, in contrast, the number of anomalies detected by RADM is always greater than the anomalies detected by the anomaly detection in UTS, that is because when the anomaly points are divided into anomaly regions, RADM can detect more anomaly points, which makes some of the anomaly regions that should be separated in the case of anomaly detection in UTS are merged together in the case of anomaly detection in MTS, so that the number of anomaly regions is reduced. In general, the average precision of RDAM is 0.706. By contrast, the average precision of HTM is 0.642.

Compared with the running time of the two experiments, we can see that the running time of RADM is about three times of the anomaly detection algorithm based on HTM, even so, RADM also has a high computational efficiency. That is because for every 4000 time series it only costs about 65 s, which has been able to meet the requirements of real-time anomaly detection.

## 6. Conclusions

Anomaly detection is an important research direction. Even though one of the terminal nodes performs anomalously, it will gradually expand the impact and threaten the entire system. RADM algorithm proposed in this paper, which combines HTM algorithm and BN effectively, can be applied to the anomaly detection in MTS in complex system. Compared to UTS, the analysis for MTS can detect anomalies in the system effectively. First of all, we use HTM algorithm to detect anomalies in UTS and get fine detection results as well as excellent time performance. Secondly, we combine HTM algorithm and BN together to perform anomaly detection in MTS effectively without dimension reduction. Some anomalies that be left out in UTS can be detected by this means and detection accuracy is improved. Lastly, in order to improve the efficiency of the algorithm, we introduce the concept of health factor α to describe whether the system is healthy or not. This method can greatly improve the performance of the algorithm in the health system. Extensive simulation results show that using RADM algorithm to perform anomaly detection in MTS can achieve better result than just in UTS. Furthermore, it will remarkably improve the performance of real-time anomaly detection in many domains. Our future work will include optimizing our algorithm further and improving the detection accuracy. We also attempt to build stronger correlations between multiple variables using other models and apply our algorithms to other areas.

## Figures and Tables

**Table 1:** List of the tail probability values.

Tail Probability Elements	Values
Q[0]	0.500000000
Q[1]	0.460172163
Q[2]	0.420740291
⋯	⋯
Q[68]	0.000000000005340
Q[69]	0.000000000002653
Q[70]	0.000000000001305

**Table 2:** The discrete values of MTS.

Time Series	Threshold Intervals	Discrete Values
Input	0-0.890401416	1
	0.890401416-0.997300202	2
	0.997300202-0.99998220767	3
	0.99998220767-1	4
Output	Normal	1
	Anormal	2

**Table 3:** Performance comparison.

Group	Sequence Length	Algorithm	Number of Anomaly Regions	Precision
1	4806	HTM	18	0.613
		RADM	27	0.675
2	4688	HTM	22	0.667
		RADM	22	0.724
3	4317	HTM	27	0.603
		RADM	27	0.632
4	4216	HTM	21	0.659
		RADM	22	0.757
5	4813	HTM	32	0.724
		RADM	26	0.775
6	4127	HTM	17	0.655
		RADM	15	0.710
7	4342	HTM	14	0.584
		RADM	14	0.656
8	4334	HTM	20	0.653
		RADM	22	0.749
9	4656	HTM	21	0.622
		RADM	25	0.695
10	4357	HTM	28	0.707
		RADM	27	0.778
11	4442	HTM	16	0.575
		RADM	20	0.644
12	4553	HTM	22	0.643
		RADM	22	0.686
